# Effect of Traditional Japanese Medicine, Daikenchuto (TJ-100) in Patients With Chronic Constipation

**DOI:** 10.4021/gr219w

**Published:** 2010-07-20

**Authors:** Akira Horiuchi, Yoshiko Nakayama, Naoki Tanaka

**Affiliations:** a Digestive Disease Center, Showa Inan General Hospital, Komagane, Japan; b Department of Pediatrics, Shinshu University School of Medicine, Japan; c Department of Metabolic Regulation, Shinshu University Graduate School of Medicine, Matsumoto, Japan

**Keywords:** Daikenchuto (TJ-100), Chronic constipation, Irritable bowel syndrome

## Abstract

**Background:**

This study was to compare the effect of a stimulant laxative alone and in combination with traditional Japanese medicine Daikenchuto (TJ-100) in improving stool frequency and in alleviating bloating and abdominal pain in patients with chronic constipation.

**Methods:**

Twenty-two patients with chronic constipation who required sennoside (24 - 60 mg daily) were allocated to two groups for treatment with 7.5 g /day (N = 14) or with 15 g/day (N = 8) of TJ-100. The study period was 12 weeks and consisted of 4 weeks (pretreatment phase) before the administration of TJ-100, 6 weeks (treatment phase) for the administration of TJ-100, and 2 weeks (washout period) after cessation of TJ-100. The bowel movement frequency and the dose of sennoside required were recorded during the study period. Bloating and abdominal pain and gastrointestinal symptoms rating scale were evaluated at 0, 4, 6, and 8 weeks. The gas volume score was measured at 0 week and 6 weeks.

**Results:**

The addition of TJ-100 to sennoside resulted in significant improvement in bloating (P < 0.01) and abdominal pain (P < 0.05). Its effects for abdominal pain were dose-dependent. There was no significant change in frequency of bowel movements or the dose of sennoside used. The gas volume score was significantly decreased after the addition of TJ-100 (P < 0.05).

**Conclusions:**

The addition of a traditional Japanese medicine, TJ-100, reduced bloating and abdominal pain in patients with chronic constipation receiving stimulant laxatives, possibly by decreasing the bowel gas volume.

## Introduction

Constipation predominant irritable bowel syndrome (IBS) and chronic constipation are often managed with a combination of stool softeners, laxatives, antispasmodics, diet changes and antidepressants [1-3]. However, uncomfortable abdominal symptoms such as bloating and abdominal pain often continue even when the stool frequency is improved by taking the above drugs. In addition, a stimulant laxative itself may induce abdominal pain.

In Japan a traditional herbal medicine Daikenchuto (Tsumura, Tokyo, Japan) has been commonly used for uncomfortable abdominal symptoms in patients with IBS and chronic constipation. Daikenchuto extract powder is manufactured as an aqueous extract containing processed ginger (Zingiber Officinale ROSCOE, rhizome), ginseng (Araliaceae, Panax ginseng C.A. MEYER, radix), and zanthoxylum fruit (Rutaceae, Zanthoxylum piperitum DE CADOLLE) in the ratio of 5:3:2. To produce the product, the three medical herbs are extracted with purified water at 95°C for 1 hour. The extract solution is then separated from the non-soluble waste and concentrated by removing water under reduced pressure. Finally, spray-drying is used to produce a dried extract powder. The yield of the extract is approximately 12.5%. The final product Daikenchuto (TJ-100) is prepared by mixing Daikenchuto extract powder and maltose syrup powder at a ratio of 1:8.

TJ-100 has been reported to alleviate constipation in children and Parkinsonian patients [4, 5] and to be effective in postoperative ileus [6]. The mechanism of its action of TJ-100 is thought to be due to an increase in gastrointestinal motility [7-13]. This study compared the effect of a stimulant laxative alone and in combination with TJ-100 for improving stool frequency and/or alleviating bloating and abdominal pain in patients with chronic constipation.

## Patients and Methods

### Patients

This study was done between January 2008 and December 2008. The study was approved by the institutional review board of Showa Inan General Hospital. All patients who were enrolled in this study fulfilled the following criteria: 1) they were taking a stimulant laxative, 24-60 mg/day of sennosides (Pursennid®, Novartis, Tokyo) for at least three months; 2) they had abdominal symptoms including bloating and abdominal pain; 3) without the laxatives, the bowel movements were less than 3 times per week; 4) colonoscopy was normal; and 5) they had no history of abdominal surgery.

The study period was 12 weeks, consisted of 4 weeks (pretreatment phase) before the administration of TJ-100, 6 weeks (treatment phase) for the administration of TJ-100, and 2 weeks (washout period) after cessation of TJ-100. The dose of TJ-100 used in this study was either 7.5 g/day or 15 g/day.

After signing informed consent, all patients fulfilling the entry criteria were randomly allocated via a simple randomization table to one of the two active treatment groups 7.5 g/day or 15 g/day of TJ-100. Sennoside (24 - 60 mg) was continued throughout the study period. The same physician managed each patient.

The bowel movement frequency and Bristol scale score were recorded daily by each patient during the study period and were measured at 0, 4, 6 and 8 weeks as the mean value per day for the week evaluated. Both bloating and abdominal pain were evaluated using visual analogue scale (VAS) score (0 - 100; 0, none). Abdominal symptoms were evaluated on the Gastrointestinal Symptoms Rating Scale (GSRS) one a scale of 1 to 5 (1 = none). The gas volume score (gas volume) was measured at 0 week and 6 week using the method described by Koide et al [14] (see below).

Further, for inclusion in our study, the patients had to be neurologically intact with no known underlying psychiatric illness. Urinalysis and hematological evaluation of total and differential white blood counts, electrolytes, liver function tests, and thyroid function tests had to be negative in all the patients considered for inclusion in the study.

### Gas volume

Before and after this administration, gas volume was calculated from a plain abdominal radiogram [14]. Briefly, plain abdominal radiographs in the supine position taken in the fasting state in the morning were digitized and transmitted to the computer. After the region of the bowel gas was identified, its outline was traced on the monitor. The total area for the region of bowel gas (gas in stomach was excluded) was determined as the pixel value on images by using Image J 1.40 g. The ratio of the quantity of bowel gas to the pixel value in the region surrounded by a horizontal line tangential to the suprasymphysary margin, a horizontal line tangential to the uppermost diaphragm and the most lateral line tangential to the right and left costal arches was defined as gas volume score (GVS).

### Analysis

The outcome measures were frequency of bowel movements per day and the level of bloating and abdominal pain and GSRS and GVS. The group medians (interquartile ranges) were obtained by taking into account all the individual daily values in that group. The individual patients were used as their own controls for their respective pre- and post-treatment values. The Wilcoxon signed-rank test was used for repeated measurements on a single sample. Differences were considered significant if the p value was less than 0.05. All statistical evaluation was performed by using SPSS version 12.0J software (SPSS Japan Inc., Tokyo, Japan).

## Results

A total of 28 patients were screened and 22 patients fulfilled all entry criteria and were enrolled in the study. Twenty-two patients were assigned to 7.5 g/day group of TJ-100 and 15 g/day group of TJ-100. As shown in Table 1, both groups were similar in age, gender and mean duration of symptoms prior to diagnosis. In both treatment groups, the bowel movement frequency was not significantly changed by the administration of TJ-100 (Table 2). The dose of sennoside used and Bristol stool form scale showed no significant changes in the two groups. With regard to bloating, a significant improvement in the median VAS score was obtained after the treatment (Table 3). With abdominal pain, a significant decrease in the median VAS score was obtained at 4, 6 and 8 weeks in 15 g /day group 32 (0 week) vs. 9 (4 weeks), (p = 0.02) and the effects of TJ-100 were obtained dose-dependently (Table 4). In both treatment groups the whole GSRS scores were improved after the administration of TJ-100 at 4, 6 and 8 weeks (Table 5). With 7.5 g/day and 15 g/day treatment groups, the median GVS was significantly decreased from 0.049 and 0.042 (0 week, p = 0.02) to 0.040 and 0.036 (6 week, p = 0.016), respectively.

**Table 1:** Demographic and Baseline Characteristics of 22 Patients With Chronic Constipation

Group	7.5 g/day (N = 14)	15 g/day (N = 8)	
Gender (male/female)	8/6	4/4	0.69
Age (years) (mean ± SD)	69.2 ± 13	68.9 ± 16	0.86
Mean duration of symptoms prior to diagnosis (month)	18	19	0.88

**Table 2:** Bowel Movement Frequency

	TJ-100			
	7.5 g/day (N = 14)	p value	15g/day (N = 8)	p value
0 week	0.7 (0.3)		0.9 (0.4)	
4 weeks	0.9 (0.2)	0.05	0.9 (0.3)	0.25
6 weeks	0.9 (0.4)	0.66	0.9 (0.2)	0.09
8 weeks	0.9 (0.3)	0.34	1 (0.2)	0.63

The frequency of bowel movement at 0, 4, 6 and 8 week is shown as the median/day of the week. Data are shown as median (interquartile range). p value shows the difference between 0 week and 4, 6, or 8 weeks

**Table 3:** Abdominal Bloating

	TJ-100			
	7.5 g/day (N = 14)	p value	15 g/day (N = 8)	p value
0 week	55 (27)		69 (13)	
4 weeks	20 (21)	0.006	35 (16)	0.007
6 weeks	17 (18)	0.007	21 (14)	0.007
8 weeks	19 (17)	0.005	15 (8)	0.007

The degree of bloating before and after the treatment of two doses of TJ-100 (7.5 g/day and 15 g/day) is shown using visual analogue scale (VAS) score (0-100; 0, none).

Data are shown as median (interquartile range). p value shows the difference between 0 week and 4, 6, or 8 weeks.

**Table 4:** Abdominal Pain

	TJ-100			
	7.5 g/day (N = 14)	p value	15 g/day (N = 8)	p value
0 week	20 (31)		32 (25)	
4 weeks	16 (19)	0.04	9 (12)	0.02
6 weeks	13 (16)	0.12	7 (12)	0.02
8 weeks	19 (14)	0.13	7 (6)	0.03

The degree of abdominal pain before and after the treatment of two doses of TJ-100 (7.5 g/day and 15 g/day) is shown using visual analogue scale (VAS) score (0-100; 0, none).

Data are shown as median (interquartile range). p value shows the difference between 0 week and 4, 6, or 8 weeks.

**Table 5:** Gastrointestinal symptoms rating scale (GSRS)

	TJ-100			
	7.5 g/day (N = 14)	p value	15 g/day (N = 8)	p value
0 week	2.6 (0.6)		2.8 (0.5)	
4 weeks	2.2 (0.5)	0.002	2.3 (0.3)	0.008
6 weeks	2.2 (0.5)	0.0002	2.3 (0.4)	0.008
8 weeks	2.3 (0.5)	0.003	2.2 (0.4)	0.008

The change of gastrointestinal symptoms rating scale (GSRS) (1-5; 1, none) before and after the treatment of two doses of TJ-100 (7.5 g/day and 15 g/day) is shown.

Data are shown as median (interquartile range). p value shows the difference between 0 week and 4, 6, or 8 weeks.

No obvious clinical side effects such as dehydration, vomiting, rectal bleeding, weight loss or headache were observed during the treatment in any patients.

## Discussion

The purpose was to investigate whether the addition of TJ-100 to the stimulant laxative improved stool frequency and alleviate bloating and abdominal pain in patients with chronic constipation. There has no such published data reported previously. Therefore, we used patients as their own controls in a pre-treatment vs. post-treatment model and examined the dose-dependent effects using two doses of TJ-100. This study demonstrated that the combination of the stimulant laxative and the traditional Japanese medicine, TJ-100 led to a significant reduction in bloating and abdominal pain, although the bowel movement frequency and Bristol scale score were not significantly changed. As TJ-100 was effective for constipation itself in children and in Parkinsonian patients [4, 5], the reason for the discrepancy of the results between this study and the previous studies is unknown.

Sennosides, the main ingredients of senna, can be classified as anthranoid-containing herbal laxatives. They are extracted from senna leaves and pods from Cassia angustifolia and Cassia acutifolia, subshrubs growing in India and Egypt [15]. Because of their natural origin, sennosides are often considered to be innocuous laxatives and thus frequently used and abused as self-medications for constipation. Chronic use of sennoside laxatives, however, induces uncomfortable abdominal symptoms in spite of the increased frequency of bowel movements.

In this study these patients with chronic constipation had concomitant abdominal pain and bloating, although it is unknown whether the symptoms arose in relation to being constipated or represented IBS or even whether the group was a mixture of both. Therefore, it remains unknown whether the TJ-100 improved the pain and bloating of a functional bowel disorder or provided benefit to counteract the side effects of the stimulant laxative. Subsequent studies will need to study both groups separately. The addition of TJ-100 to the stimulant laxative led not only to reduction in bloating, but also to reduction in abdominal pain. This suggests that it may be possible to use combination therapy to achieve relief of constipation and at the same time alleviate bloating and abdominal pain.

In this study it seems that the 7.5 g/day dose and the 15 g/day dose were equally effective for bloating, the GSRS and the gas volume as measured by abdominal X-rays. The beneficial effect of TJ-100 might have resulted from directly stimulating colonic motility and decreasing the bowel gas volume. This result confirmed the enhanced effects of TJ-100 on colonic motility in the previous studies [11-13]. Of interest, TJ-100 showed a dose-dependent effect on abdominal pain. This beneficial effect may be due to a mechanism in addition to decreasing the bowel gas volume.

One study limitation was that there was no placebo-controlled comparison. The data were analyzed using patients as their own controls and as shown in Tables 3 to 5, the values did not return to baseline after cessation of TJ-100. The post treatment phase was only 2 weeks and may not have been sufficient for a return to baseline. Other possibilities are that the use of TJ-100 resulted in a prolonged change in bowel function in these patients or that the changes noted from the initial baseline reflected a placebo response such as a regression to the mean [16]. Further studies with longer duration of follow-up and containing a placebo are needed to further understand these results.

## Abbreviations

TJ-100: Daikenchuto
GSRS: gastrointestinal symptoms rating scale
IBS: irritable bowel syndrome
GVS: gas volume score
